# Interactive effects of locus coeruleus structure and catecholamine synthesis capacity on cognitive function

**DOI:** 10.3389/fnagi.2023.1236335

**Published:** 2023-09-06

**Authors:** Hsiang-Yu Chen, Jourdan H. Parent, Claire J. Ciampa, Martin J. Dahl, Dorothea Hämmerer, Anne Maass, Joseph R. Winer, Renat Yakupov, Ben Inglis, Matthew J. Betts, Anne S. Berry

**Affiliations:** ^1^Department of Psychology, Brandeis University, Waltham, MA, United States; ^2^Center for Lifespan Psychology, Max Planck Institute for Human Development, Berlin, Germany; ^3^USC Leonard Davis School of Gerontology, University of Southern California, Los Angeles, CA, United States; ^4^Psychological Institute, University of Innsbruck, Innsbruck, Austria; ^5^Deutsches Zentrum für Neurodegenerative Erkrankungen, Magdeburg, Germany; ^6^Helen Wills Neuroscience Institute, University of California, Berkeley, Berkeley, CA, United States; ^7^Henry H. Wheeler Jr. Brain Imaging Center, University of California, Berkeley, Berkeley, CA, United States; ^8^Institute of Cognitive Neurology and Dementia Research, Otto-von-Guericke University Magdeburg, Magdeburg, Germany; ^9^Center for Behavioral Brain Sciences, Otto-von-Guericke University Magdeburg, Magdeburg, Germany; ^10^Lawrence Berkeley National Laboratory, Berkeley, CA, United States

**Keywords:** locus coeruleus, catecholamine synthesis capacity, structural magnetic resonance imaging, positron emission tomography, partial least squares correlation

## Abstract

**Background:**

The locus coeruleus (LC) produces catecholamines (norepinephrine and dopamine) and is implicated in a broad range of cognitive functions including attention and executive function. Recent advancements in magnetic resonance imaging (MRI) approaches allow for the visualization and quantification of LC structure. Human research focused on the LC has since exploded given the LC’s role in cognition and relevance to current models of psychopathology and neurodegenerative disease. However, it is unclear to what extent LC structure reflects underlying catecholamine function, and how LC structure and neurochemical function are collectively associated with cognitive performance.

**Methods:**

A partial least squares correlation (PLSC) analysis was applied to 19 participants’ LC structural MRI measures and catecholamine synthesis capacity measures assessed using [^18^F]Fluoro-m-tyrosine ([^18^F]FMT) positron emission tomography (PET).

**Results:**

We found no direct association between LC-MRI and LC-[^18^F]FMT measures for rostral, middle, or caudal portions of the LC. We found significant associations between LC neuroimaging measures and neuropsychological performance that were driven by rostral and middle portions of the LC, which is in line with LC cortical projection patterns. Specifically, associations with executive function and processing speed arose from contributions of both LC structure and interactions between LC structure and catecholamine synthesis capacity.

**Conclusion:**

These findings leave open the possibility that LC MRI and PET measures contribute unique information and suggest that their conjoint use may increase sensitivity to brain-behavior associations in small samples.

## 1. Introduction

The locus coeruleus (LC) is a small cylindrical and hyperpigmented nucleus in the posterior pontine brainstem and is the brain’s major source of norepinephrine. Evidence from animal models suggests rostral-caudal topographic organization with rostral aspects projecting to the frontoparietal cortex and temporal lobe (Loughlin et al., 1986; Schwarz and Luo, 2015), and caudal aspects projecting to the cerebellum and spinal cord (Loughlin et al., 1986; Manaye et al., 1995; Schwarz and Luo, 2015). Following this topography, rostral LC is implicated in cognitive functions including attention, executive function, and memory, while caudal LC is implicated in motor and autonomic functions (Dahl et al., 2019, 2022; Liu et al., 2020; though also see Hirschberg et al., 2017; Poe et al., 2020; Veréb et al., 2023 for discussion of the substantial complexity of such functional subdivisions). Relevant to aging neuroscience, the LC is one of the earliest brain regions to accumulate abnormal tau protein and has thus been a focus of intense research interest given its possible role in the early pathophysiology of Alzheimer’s disease (Braak et al., 2011; Andrés-Benito et al., 2017; Ehrenberg et al., 2017, 2023; Jacobs et al., 2021).

Advances in specialized magnetic resonance imaging (MRI) approach for visualizing LC structure have contributed to an explosion in new research investigating the role of the LC in cognition (e.g.,Betts et al., 2017; Clewett et al., 2018; Dahl et al., 2019), neurodegenerative diseases including Alzheimer’s disease (Betts et al., 2019a; Jacobs et al., 2021; Dahl et al., 2022) and Parkinson’s disease (Doppler et al., 2021), psychopathology (Shibata et al., 2007; Calarco et al., 2022), and addiction (Biernacki et al., 2020). The MR contrast mechanisms supporting LC visualization are still debated but likely rely, at least in part, on the magnetic properties of neuromelanin in the LC neuron. Neuromelanin is a by-product of catecholamine (norepinephrine and dopamine) synthesis in the LC (Zecca et al., 2004), and may be visualized with MRI due to shortened T1 or magnetization transfer (MT) effects (see Betts et al., 2019b for discussion).

It is not known whether structural LC measures afforded through MR imaging are related to the underlying catecholaminergic synthesis capacity of the LC, which might be expected given that neuromelanin is mutually implicated in the MR contrast and catecholamine synthesis. Catecholamine synthesis capacity can be measured *in vivo* using positron emission tomography (PET) and irreversible tracer [^18^F]Fluoro-m-tyrosine ([^18^F]FMT). [^18^F]FMT is a substrate for the catecholamine synthesis enzyme aromatic amino acid decarboxylase. Our recent research has established [^18^F]FMT net tracer influx (Ki) in the LC as a useful measure for understanding individual differences in cognition in older adults (Ciampa et al., 2022a; Parent et al., 2022). While LC catecholamine synthesis capacity was not directly related to better memory, we found that higher catecholamine synthesis capacity was associated with better-than-expected memory performance in individuals with substantial temporal lobe tau burden. These interactive effects suggest that *in vivo* assessment of neurochemical function can improve models defining the neural basis of individual differences in neuropsychological performance (Ciampa et al., 2022a).

In this study, we measured individual participants’ LC structure using MT-MRI and catecholamine synthesis capacity using [^18^F]FMT-PET. We applied partial least squares correlation analysis (PLSC), which is a powerful tool for detecting latent associations in small samples (McIntosh and Lobaugh, 2004; Krishnan et al., 2011; Dahl et al., 2022), to test relationships between LC MRI and PET measures in a sample of 19 adults (20–84 years old). Next, we tested associations between LC measures and cognitive performance using a battery of neuropsychological assessments, which included standard measures of memory, executive function, and processing speed. To preview our results, we found no direct relationship between LC structure and catecholamine synthesis capacity measures. However, we found LC-cognition associations arising from independent contributions of LC structure and interactions between LC structure and catecholamine synthesis capacity. Consistent with frameworks describing rostral versus caudal LC anatomical projections, LC associations with neuropsychological performance were driven by rostral LC structure and neurochemical function and were not observed for caudal LC measures.

## 2. Methods

### 2.1. Participants

Nineteen participants (11 males, 9 young adults aged: 20–29 years [mean ± SD = 24.78 ± 2.21], and 10 older adults: 65–84 years [mean ± SD = 77.4 ± 5.76]) with normal or corrected-to-normal vision participated in the study. Participants were part of the Berkeley Aging Cohort Study and scored at least 25 points on the Mini-Mental State Exam (mean ± SD = 29.26 ± 1.10). Participants did not have a neurological, psychological, or psychiatric disorder and did not take medication affecting cognition. Each participant completed a neuropsychological battery, MRI, and PET sessions on separate days (time difference between neuropsychological testing and MRI: mean ± SD = 253 ± 345 days; time difference between MRI and PET: mean ± SD = 259 ± 353 days; time difference between neuropsychological testing and PET: mean ± SD = 210 ± 116 days). Written informed consent was obtained from all participants. The study was approved by the Lawrence Berkeley National Laboratory Institutional Review Board of the University of California, Berkeley. A subset of [^18^F]FMT PET data (10 out of 19 participants, mean age ± SD = 77.40 ± 5.76) contributed to a report describing relationships between LC catecholamine synthesis capacity and temporal lobe tau pathology (Ciampa et al., 2022a). LC structure analysis and the associations between the interactive effects of LC structure and LC catecholamine synthesis capacity and cognitive performance here have not been reported elsewhere.

### 2.2. Acquisition of structural MRI data and LC intensity assessment

Scans were acquired on a 3 T Siemens Magnetom Trio Tim whole-body scanner with a standard 32-channel radiofrequency head coil for signal reception. A high-resolution T1-weighted anatomical image was acquired using a magnetization-prepared rapid gradient echo (MPRAGE) sequence for each participant. The parameters for the T1-weighted MPRAGE were as follows: voxel size = 1 × 1 × 1 mm, 160 slices, TR = 2,300 ms, TE = 2.98 ms, flip angle = 15°, sagittal plane, acquisition matrix = 256 × 240 × 160. An additional 3D magnetization transfer-weighted (MTw) acquisition was acquired to capture the entire pons region. Protocol parameters were as follows: voxel size = 0.78 × 0.78 × 1 mm, TR = 45 ms, TE = 5.36 ms, flip angle = 23°, bandwidth = 130 Hz/pixel, acquisition matrix = 176 × 256 × 64. The axial 3D block was oriented parallel to the floor of the 4th ventricle during acquisition and only covered the brainstem region. The duration of the MTw brainstem scan was 5:44 min.

To improve the visualization in the LC region for further manual segmentation, each participant’s MTw image was upsampled to an equivalent resolution of 0.39 × 0.39 × 0.5 mm using a *sinc* function in Matlab as previously described (Betts et al., 2019b). RF-bias corrected with N4-ITK sinc interpolated MTw images were standardized (to a study-wise space) by way of a parallel routine for template calculation provided in ANTs v2.1. Simultaneous co-registrations were driven by a cross-correlation minimization performed over three resolutions with a maximum of 90 iterations at the coarsest level, 30 at the next coarsest, and 90 at full resolution; the template update step size was set to 0.1 mm. One rigid-then-affine iteration was followed by eight full runs of the above non-linear multi-resolution routine to ensure stable convergence. The spatial standardization needed to generate the study-wise template was achieved through the warp composition of the above transformations and third-order b-spline interpolation. The LC and reference masks delineated in template space were warped to each participant in the study-wise template using the ‘WarpImageMultiTransform’ command and nearest neighbor interpolation in ANTs v2.1.

**Figure 1:**
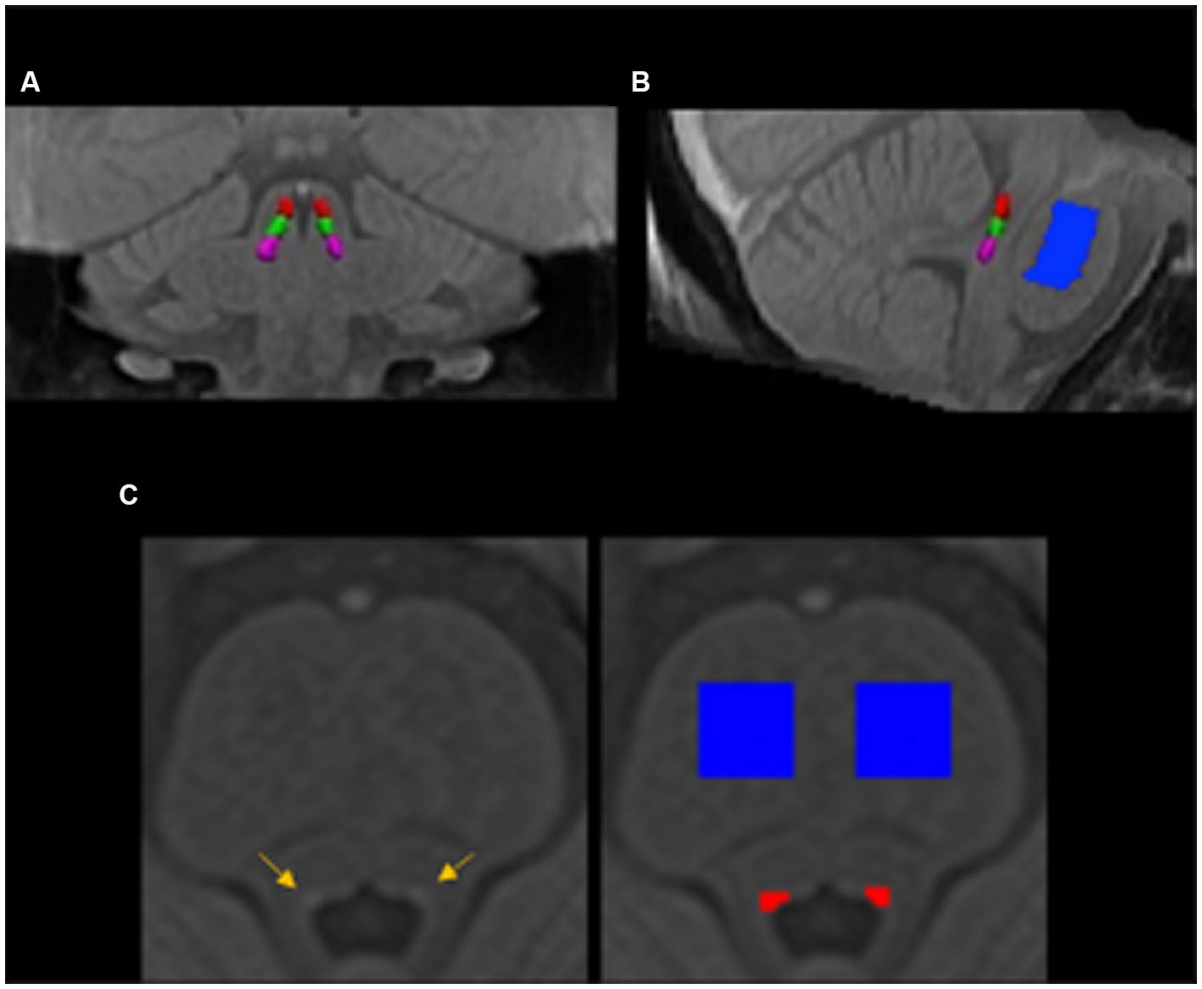
The locus coeruleus and pontine reference masks in the group template space in the coronal **(A)**, sagittal **(B)**, and axial **(C)** view. The locus coeruleus (yellow arrows) and pontine reference (blue) regions of interest were manually segmented in the group brainstem template and then warped back to individual’s native space. The delineated locus coeruleus region was equally split into rostral (red), middle (green), and caudal (violet) parts in the rostrocaudal dimension.

The LC was manually segmented on the MTw study-wise template using the software package ITK-SNAP (Yushkevich et al., 2006) as previously described (Betts et al., 2017). Briefly, the most superior LC slice in the axial plane was defined immediately after the inferior boundary of the interpeduncular fossa at the level of the inferior colliculus. The LC mask was delineated from rostral to caudal direction at each axial slice across 20 slices (20 mm). In addition, a pontine reference region (20 × 20 × 20 voxels) was segmented in each hemisphere in the axial plane. The reference masks started at the slice corresponding to the most rostral aspect of the delineated LC. The LC and reference masks delineated in template space were warped to each participant in the study-wise template using the ‘WarpImageMultiTransform’ command and nearest neighbor interpolation in ANTs v2.1. Given the use of a group-level LC mask, we performed quality assurance checks on all individual participant data. The LC masks in each participant’s MT-MRI image were visually inspected and corrected (three out of 19 participants) to ensure no spurious voxels in the 4^th^ ventricle or gap in the rostrocaudal axis of the LC. Each participant’s LC mask was split into three equal segments (rostral, middle, and caudal; Figure 1) along with the slices from rostral to caudal direction of the LC. Hence, the LC-MRI contrast ratio was then calculated for each segment on each hemisphere as follows:


$$LC_{ratio}=\frac{\left(S_{LC}-S_{Ref}\right)}{S_{Ref.}}$$

where *S_LC_* denotes the mean signal intensity in the masked LC region, and *S_Ref_* indicates the mean signal intensity in the masked pontine reference region.

### 2.3. Acquisition of [^18^F]FMT PET data and LC [^18^F]FMT assessment

Participants underwent [^18^F]FMT PET using a Siemens Biograph Truepoint 6 PET/CT. The extended description of [^18^F]FMT PET acquisition and preprocessing analysis is described in our previous work (Ciampa et al., 2022a). Briefly, dynamic acquisition frames were obtained over a 90-min duration in 3D mode (25 frames total: 5 × 1, 3 × 2, 3 × 3, 14 × 5 min). Frames were realigned, coregistered, and resliced to individual participants’ structural MRI (MPRAGE) images and then converted to whole-brain images (Ki_vis_) using the primary visual cortex (lingual gyrus and cuneus) as the reference region (Patlak and Blasberg, 1985). Ki_vis_ represents the amount of tracer accumulated in the brain relative to the visual cortex reference region. In addition, structural (MPRAGE) templates in young and older adult groups were separately generated in Montreal Neurological Institute (MNI; 1 mm) space using Statistical Parametric Mapping software’s DARTEL (SPM12; Wellcome Trust Center for Neuroimaging, London, UK). Whole-brain Ki_vis_ images were normalized to the DARTEL template in the MNI space (Ciampa et al., 2022a) for further analyses.

To obtain [^18^F]FMT Ki_vis_ measures in the comparable three segments of the LC, we coregistered the delineated LC mask in the group brainstem template described above to the Montreal Neurological Institute (MNI; 1 mm) space using the nearest neighbor interpolation and then split the LC mask into equal rostral, middle, and caudal segments. The mean [^18^F]FMT Ki_vis_ in each masked LC segment was computed for each participant.

### 2.4. Neuropsychological tests

To evaluate basic cognitive function, participants underwent a battery of neuropsychological tests including the Mini-Mental State Examination (MMSE), Wechsler Adult Intelligence Scale III (WAIS-III), Wechsler Memory Scale IV (WMS-IV), Wisconsin Card Sorting (WCS) task, California Verbal Learning Test (CVLT), Trail Making task, Verbal Fluency task, and Stroop task. Table 1 in the Results section depicts the details of each neuropsychological test and scoring.

**Table 1:** Description of neuropsychological test and scoring and correlation coefficients with the latent LC brain measures.

Cognitive domain	Neuropsychological test	Scoring	Correlation coefficient	*p-*value
General cognition	MMSE	A score of 25 (out of 30) or higher is identified as cognitively normal.	0.29	0.28
Cognitive flexibility (set-shifting)	WCS total correct	Number of correct responses	0.09	0.76
	WCS perseverative responses	Number of responses an individual continues with the same response strategy	−0.16	0.56
	WCS non-preservative errors	Number of non-preservative errors	−0.07	0.79
	WCS perseverative errors	Number of responses an individual continues with the same response strategy following a rule switch	−0.10	0.73
	WCS % perseverative errors	The total number of perseverative errors divided by the number of trials administered	−0.10	0.73
	WCS categories	Number of categories	0.36	0.17
	WCS % conceptual level responses	Percentage of correct responses occurring in runs of three or more	0.11	0.69
	WCS failure to maintain set	Number of responses an individual changes response strategies despite the rule remaining the same	−0.48	0.06
	Trail making A	Time in seconds an individual spends to finish trail A	−0.42	0.11
	Trail making B	Time in seconds an individual spends to finish trail B	−0.50	0.046*
Cognitive stability (working memory; inhibition)	Forwards digital span (WAIS-III)	Number of correct responses for digits recall forward	0.39	0.14
	Backward digital span (WAIS-III)	Number of correct responses for digits recall backward	0.53	0.04*
	Stroop	Time difference in milliseconds between neutral and incongruent trials	0.04	0.87
Processing speed	Digit symbol (WAIS-III)	Number of correct responses	0.50	0.047*
	Mental control (WMS IV)	Number of correct responses	0.17	0.53
Verbal memory	CVLT trial 1–5 free recall total	Number of correct recall items after reading the target list in 5 trials	0.31	0.25
	CVLT short delay free recall	Number of correct recall items in the target list without any cues after reading the distractor list	0.35	0.18
	CVLT short delay cued recall	Number of correct recall items in the target list with any cues after reading the distractor list	0.07	0.81
	CVLT long delay free recall	Number of correct recall items in the target list without any cues after 20 min	0.33	0.21
	CVLT long delay cued recall	Number of correct recall items in the target list with cues after 20 min	0.11	0.69
	CVLT total intrusions	Number of recall items that are not in the target list	0.33	0.22
	CVLT total repetitions	Number of repeated recall items	−0.24	0.38
	CVLT recognition hits	Number of items that are in the target list an individual recognizes	−0.28	0.29
	CVLT recognition false positives	Number of items that are not in the target list but an individual claims	0.37	0.16
	Logical memory (WMS IV)	Number of correct recalls	0.47	0.07
	Verbal paired associates (WMS IV)	Number of correct recall word-pairs.	0.33	0.22
Visual memory	Visual reproduction I recall total (WMS IV)	Sum of total performance scores	0.46	0.07
	Visual reproduction II recall total (WMS IV)	Sum of total performance scores	0.23	0.39
	Visual reproduction recognition total (WMS IV)	Sum of total performance scores	0.27	0.32
	Visual reproduction % retention (WMS IV)	Sum of total scores in visual reproduction II divided by Sum of total scores in visual reproduction I	0.02	0.94
Language (Verbal fluency)	Phonemic fluency	Number of words starting with F, A, or S	0.11	0.69
	Category fluency	Number of words that are categorized as vegetables or animals	−0.02	0.95

MMSE, mini-mental state examination; WCS, Wisconsin card sorting task; WAIS-III, Wechsler adult intelligence scale III; WMS IV, Wechsler memory scale IV; CVLT, California verbal learning test. *indicates significance with *p* < 0.05.

### 2.5. Statistical analysis

**Figure 2:**
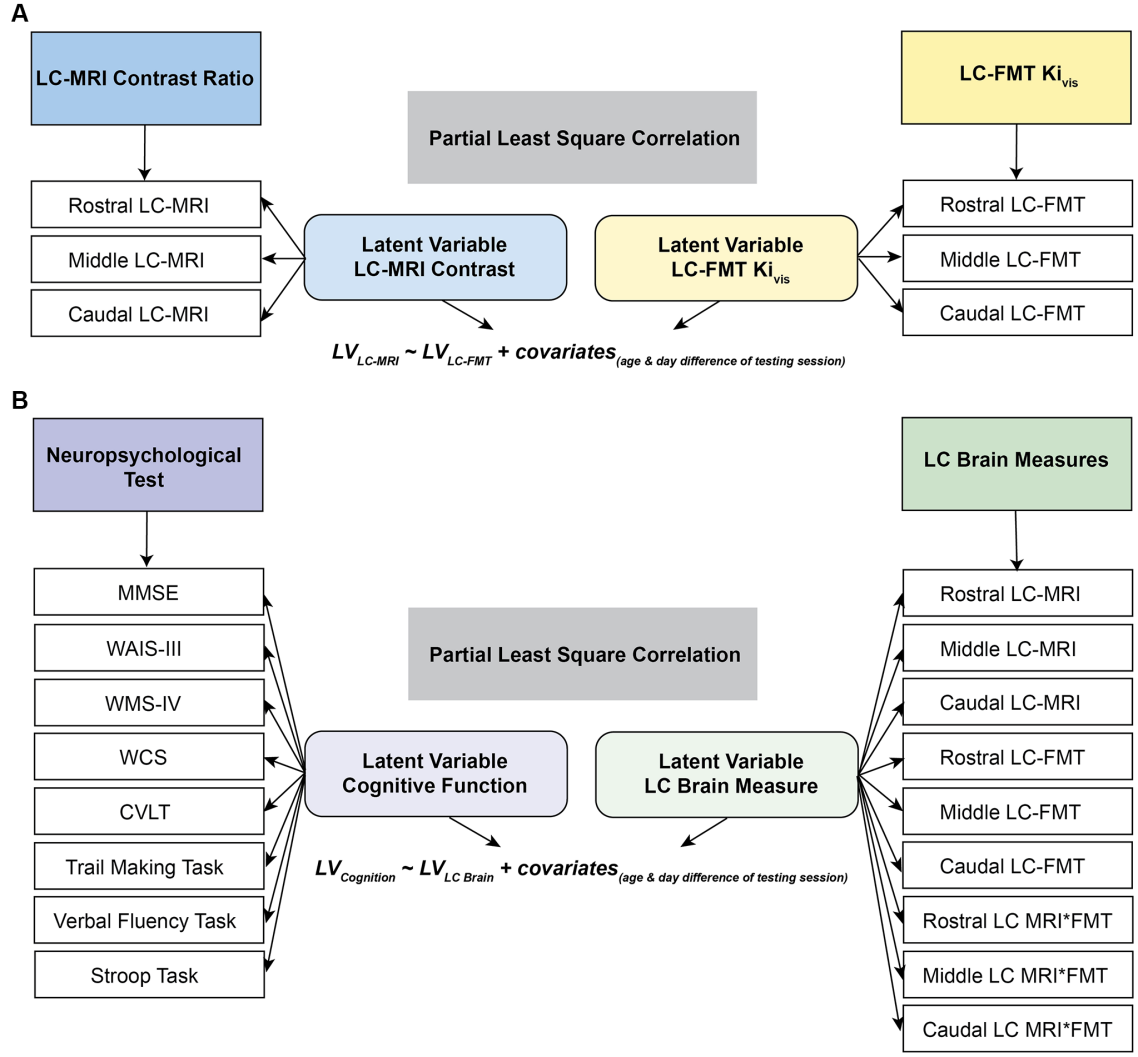
Schematic representation of partial least squares correlation analysis (PLSC). The correlation matrix between the LC-MRI contrast ratios and LC MFT Ki_vis_ **(A)** or between the LC brain measures or neuropsychological performance **(B)** was subjected to singular vector decomposition. The association between the two latent variables after partialling out the chronological age and temporal day difference between cognitive and neuroimaging sessions represents the pattern of interindividual differences in LC-MRI contrast ratios that shares the largest amount of variance with interindividual differences in overall LC [^18^F]FMT Ki_vis_ **(A)** or that of interindividual differences in the LC brain measures that share the maximal covariance with interindividual differences in overall neuropsychological performance **(B)**. MMSE, mini-mental state examination; WCS, Wisconsin card sorting task; WAIS-III, Wechsler adult intelligence scale III; WMS IV, Wechsler memory scale IV; CVLT, California verbal learning test.

To investigate the multivariate association between LC structure and LC catecholamine synthesis capacity as well as their relationship with cognitive function, we used PLSC analysis (McIntosh and Lobaugh, 2004; Krishnan et al., 2011; Dahl et al., 2022). First, the correlation matrix (Eq. 1a) between LC-MRI contrast ratios (3 segments × 19 participants) and LC [^18^F]FMT Ki_vis_ (3 segments × 19 participants) was decomposed into three matrices using a singular value decomposition (SVD). The SVD can be denoted as follows:


(1a) $$R=Y_{LC-FMT}{}^{T}X_{LC-MRI}$$

(1b) $$SVD\left[R_{\left(X_{LC-MRI},Y_{LC-FMT}\right)}\right]=U_{LC-FMT}SV_{LC-MRI}$$

(1c) $$LV_{LC-MRI}=X_{LC-MRI}V_{LC-MRI}$$

(1d) $$LV_{LC-FMT}=Y_{LC-FMT}U_{LC-FMT}$$

where *X* and *Y* denote the datasets of LC-MRI contrast ratios and LC [^18^F]FMT Ki_vis_, respectively, and *R* indicates Pearson’s correlation matrix between LC-MRI contrast ratios and LC [^18^F]FMT Ki_vis_ subjected to *SVD*. All the datasets (i.e., *X_LC-MRI_* or *Y_LC-FMT_*) were *z*-scored across all participants before applying *SVD*. *U* and *V* are the singular vectors (the saliences) that represent the profiles of LC-MRI contrast and LC [^18^F]FMT Ki_vis_, respectively, to best characterize *R*. *S* is a diagonal matrix of singular values. Based on this matrix decomposition, a pair of latent variables (*LV*) was extracted to denote a linear combination of each original dataset (i.e., *X_LC-MRI_* or *Y_LC-FMT_*) and its correspondingly singular vector (i.e., *V_LC-MRI_* or *U_LC-FMT_* in Eqs. 1c or 1d). The association between the two latent variables optimally expresses (in the least squares sense) the pattern of interindividual differences in LC-MRI contrast ratios that shares the largest amount of variance with interindividual differences in overall LC [^18^F]FMT Ki_vis_ (Figure 2A).

Similarly, to express the association between LC brain measures and cognitive function, a Pearson’s correlation matrix (Eq. 2a) between the LC brain measures (LC-MRI contrast ratios, LC [^18^F]FMT Ki_vis_, and their interaction using the product of the two measures; 9 segments × 19 participants) and neuropsychological scores (33 neuropsychological tests × 19 participants) was subjected to *SVD*. Two latent variables were extracted to denote the linear combinations of original datasets (i.e., *X*_*LC* brain_ or *Y_Neuropsy_*) and their correspondingly singular vector (i.e., *V*_*LC* brain_ or *U_Neuropsy_* in Eqs. 2c or 2d). The relationship between the two extracted latent variables reflects the pattern of interindividual differences in the LC brain measures that share the maximal covariance with interindividual differences in overall neuropsychological performance (Figure 2B).


(2a) $$R=Y_{neuropsy}{}^{T}X_{LC\;brain}$$

(2b) $$SVD\left[R_{\left(X_{LC\;brain},Y_{Neuropsy}\right)}\right]=U_{Neuropsy}SV_{LCbrain}$$

(2c) $$LV_{LC\;brain}=X_{LC\;brain}V_{LC\;brain}$$

(2d) $$LV_{Neuropsy}=Y_{Neuropsy}U_{Neuropsy}$$

Because age and time difference between neuroimaging sessions (see detailed information in 2.1 Participants section) may moderate cognitive functions measured by the battery of neuropsychological tests, the associations between the extracted latent variables were examined across all participants after partialling out chronological age and temporal differences between cognitive and neuroimaging sessions. The significance of the extracted latent variables was evaluated using a permutation test (by randomly re-ordering the observations in the X dataset while leaving the Y dataset and re-calculating the SVD; *n* = 10,000). The reliability of the singular vectors to the latent variables was evaluated using bootstrapping (*n* = 10,000). A ratio of the singular vectors and their corresponding bootstrapped standard errors provided bootstrap ratios that can be interpreted akin to Z-scores (Dahl et al., 2022). Thus, bootstrap ratios < −1.96 and > 1.96 (akin to *Z*-scores; *p* < 0.05) were considered reliable and indicated as reliable contributions to the latent variable.

We also performed exploratory analyses with PLSC probing correlations between LC structural intensity ratios and [^18^F]FMT Ki_vis_ at the voxel-wise whole brain level as well as between LC structural intensity ratios and the [^18^F]FMT Ki_vis_ within 36 regions of interest (Desikan et al., 2006). We did not find any relationship in these additional analyses. The description of the analyses and results can be found in the Supplementary material section.

## 3. Results

### 3.1. Associations between LC structure and LC [^18^F]FMT Ki_vis_

PLSC analysis was applied to investigate the relationships between the LC structural integrity and LC catecholamine synthesis capacity. We found no association between the LC-MRI contrast ratios and LC [^18^F]FMT Ki_vis_. Four latent variables were extracted, but none reached significance (*ps* > 0.09).

### 3.2. Associations between LC brain measures and neuropsychological performance

**Figure 3:**
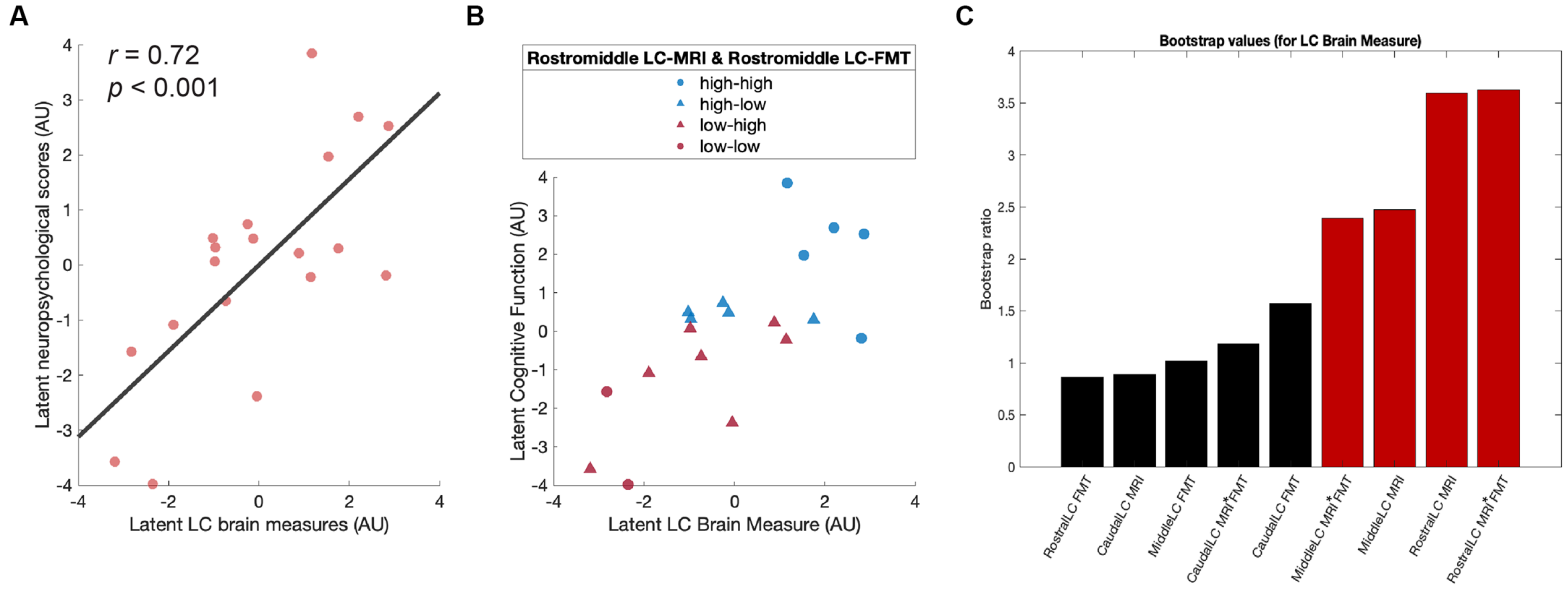
Higher LC brain measures are associated with better cognitive functions. **(A)** Scatter plot depicting the association between latent LC brain measures and latent neuropsychological scores after partialling out chronological age and temporal day difference between cognitive and neuroimaging sessions. **(B)** Scatter plot indicating the distribution of the participants split into four subgroups by the mean of the LC brain measures. **(C)** Contribution of individual LC brain measures to a PLSC latent variable (bootstrap ratios >1.96 represented in red is considered reliable). LC, locus coeruleus; PLSC, partial least squares correlation.

To test the behavioral relevance of the LC brain measures, an equivalent PLSC analysis was applied to the correlation matrix between LC brain measures (LC-MRI contrast ratios, LC [^18^F]FMT Ki_vis_ measures, and their interactions) and neuropsychological performance. We extracted the latent variables (first component *p* = 0.02) that optimally expressed the multivariate association between participants’ LC brain measures and cognitive performance (*r* = 0.72, *p* < 0.001; Figure 3A). Participants who showed higher LC brain scores exhibited better neuropsychological performance after partialling out chronological age and temporal day difference between neuroimaging sessions. Specifically, the rostral LC-MRI contrast, the interaction between the rostral LC-MRI contrast and rostral LC [^18^F]FMT Ki_vis_, the middle LC-MRI contrast, and the interaction between middle LC-MRI contrast and middle LC [^18^F]FMT Ki_vis_ were the reliable contributors (Bootstrap ratios >1.96; Figure 3C) to the latent variable of the LC brain measures. These primary contributors were correlated with neuropsychological performance related to processing speed (Digit symbol in WAIS-III: *r* = 0.50, *p* = 0.047), cognitive flexibility (trail making B: *r* = −0.50, *p* = 0.046), and cognitive stability (backward digit span: *r* = 0.53, *p* = 0.04; Table 1).

For visualization purposes, we split the sample into four subgroups based on the mean of rostral and middle LC-MRI contrast ratios and that of rostral and middle LC [^18^F]FMT Ki_vis_: high LC-MRI contrast – high LC [^18^F]FMT Ki_vis_ (high – high), high LC-MRI contrast – low LC [^18^F]FMT Ki_vis_ (high – low), low LC-MRI contrast – high LC [^18^F]FMT Ki_vis_ (low – high), and low LC-MRI contrast – low LC [^18^F]FMT Ki_vis_ (low – low). Group median values defined low-high cut points. The distribution of each group was linearly scaled in the association between the latent variables (Figure 3B). The participants in the high-high group tended to exhibit better neuropsychological performance (blue circles), while participants in the low-low group tended to exhibit worse neuropsychological performance (red circles).

## 4. Discussion

We assessed relationships between a specialized MRI measure of LC structure and a PET measure of LC catecholamine synthesis capacity and examined their conjoint associations with cognitive function. We found no direct relationship between *in vivo* measures of LC structure and underlying neurochemical function but found that interactive effects of the LC structure and catecholamine synthesis capacity were associated with better processing speed and executive function. These findings were most pronounced for the rostral and middle segments of the LC, consistent with rostral LC’s anatomical projection patterns to the cerebral cortex (Loughlin et al., 1986; Schwarz and Luo, 2015).

This study is limited by its small sample size. While limited, we pursued these analyses given the growing attention and resources granted to research incorporating LC MRI measures, and the field’s interest in considering LC MRI to be a proxy for catecholamine function (Betts et al., 2019b; Horga et al., 2021). Since 2018, the number of papers identified on PubMed with the search terms “locus coeruleus” and “MRI” has grown from 2,653 to 4,780. The novelty of the within-participant MRI and PET measures, and the extensive neuropsychological battery available within the same individuals strengthen the potential contribution of our findings.

*In vivo* PET imaging has limited spatial resolution but can have excellent signal-to-noise ratios, and has been used previously to assess LC glucose metabolism and catecholamine markers (Coull et al., 1999; Remy et al., 2005; Ding et al., 2010; Ono et al., 2016; Brumberg et al., 2019; Liu et al., 2021). However, we know little about the relationships between LC structural measures obtained by MRI and the neurochemical function of the LC. Post-mortem validation studies suggest higher LC-MRI contrast is positively correlated with higher neuromelanin concentrations (Keren et al., 2015; Cassidy et al., 2019), but few studies investigated associations with the related neurochemical modulation such as catecholamine concentration, synthesis enzymes, transporter or receptor density, or gene expression. Thus far, only two studies have examined relationships between LC structure and PET measures of catecholamine function ([^11^C]MeNER PET to measure norepinephrine transporter density) and found no direct relationships in Parkinson’s disease patients (Sommerauer et al., 2018; Doppler et al., 2021; though Sommerauer and colleagues found a positive association in the thalamus). To our knowledge, this is the first study to report analyses of LC MRI and PET associations in healthy adults.

Higher rostral LC-MRI contrast and the interaction between rostral LC-MRI contrast and rostral catecholamine synthesis capacity were the primary measures associated with better cognitive performance. These findings specifically implicating higher rostral LC-MRI contrast (and not caudal) in better cognitive function are in close agreement with previous research (Dahl et al., 2019, 2022; Liu et al., 2020). Using PLSC analyses, we identified a significant latent association between LC neuroimaging measures and cognition that was driven by associations with better executive function (cognitive flexibility: trail making B; cognitive stability: backward digit span) and processing speed (digit symbol). Notably, associations with executive function captured LC contributions to both cognitive flexibility, which supports shifting between task sets, and cognitive stability, which supports set maintenance, distractor suppression, and inhibition (Armbruster et al., 2012). These findings are consistent with previous research implicating the LC-catecholamine system executive function (Arnsten and Li, 2005; Von der Gablentz et al., 2015; Mather et al., 2016; Liu et al., 2020) as well as recent human research mapping functional and structural connectivity of the LC to the prefrontal cortex and striatum (Zhang et al., 2016; Grueschow et al., 2022; Liebe et al., 2022). Of note, we demonstrate associations between LC neuroimaging measures and cognition in a small sample that also included young adults. Defining the functional relevance of individual differences in LC MRI contrast in younger adults is an area of active research (see Clewett et al., 2018; Bachman et al., 2022 for the discussion).

Our previous research suggests that higher catecholamine synthesis capacity is associated with better executive function in young adults (Berry et al., 2016, 2018), and is a mechanism of cognitive resilience in the older age (Ciampa et al., 2022a,b). Specifically, in older adults, interaction analyses showed that catecholamine synthesis capacity moderates relationships between tau burden and memory (Ciampa et al., 2022a) as well as between cortical atrophy and executive function (Ciampa et al., 2022b). Broadly, these findings are consistent with a possible role of higher catecholamine synthesis in “rescuing” cognitive performance in the face of age and pathology-related neuronal loss. Catecholamine systems are highly regulated and show patterns consistent with compensatory upregulation in the face of the experimental lesion (Acheson and Zigmond, 1981; Fritschy and Grzanna, 1992), or losses due to Parkinson’s disease (Moore et al., 2008; Pavese et al., 2011), Alzheimer’s disease (Hoogendijk et al., 1999; Raskind et al., 1999), and normal aging (Dejesus et al., 2001; Ciampa et al., 2022a,b). An intriguing possibility is that some older adults are able to offset losses in LC structural integrity with successful maintenance or upregulation of catecholamine synthesis. Such “uncoupling” of structure and neurochemical function may reflect a mechanism of resilience in aging and neurodegenerative disease (Weinshenker, 2018).

It should be noted that previous literature has demonstrated a quadratic relationship between the LC-MRI contrast and age (Shibata et al., 2006; Liu et al., 2020). In the present study, our small sample size constrains the possibility of investigating adult age differences (young vs. older) in the associations between the LC structural integrity and LC catecholamine synthesis capacity and their interactions with cognitive functions. Following suggestions from the reviewers, we performed an exploratory piecewise regression analysis, which did not suggest associations between LC-MRI and LC-FMT measures differed based on age groups (Fig. S1). Future studies with larger samples in each age group are needed to replicate and extend the current findings. While this study has limitations, it provides critical replication of research specifically implicating the structural integrity of the LC in cognitive function (Clewett et al., 2016; Hämmerer et al., 2018; Dahl et al., 2019, 2022), and previous findings describing moderating effects of higher catecholamine synthesis capacity (Ciampa et al., 2022a,b). The addition of PET measures may improve sensitivity to detect relationships between *in vivo* neuroimaging of the LC and behavioral performance, which may be particularly beneficial for studies in patient populations or samples with prominent intraindividual variability.

## Data availability statement

The raw data supporting the conclusions of this article will be made available by the authors, without undue reservation.

## Ethics statement

The studies involving humans were approved by Lawrence Berkeley National Laboratory Institutional Review Board of the University of California, Berkeley. The studies were conducted in accordance with the local legislation and institutional requirements. The participants provided their written informed consent to participate in this study.

## Author contributions

H-YC: methodology, formal analysis (neuroimaging and cognitive data and PLSC), visualization, writing – original draft, and writing – review and editing. JP: formal analysis (PET and cognitive data) and data curation. CC: formal analysis (PET data) and data curation. MD: methodology, software, and writing – review and editing. DH: methodology and writing – review and editing. AM and JW: conceptualization, methodology, validation, resources, and writing – review and editing. RY: formal analysis (MRI data) and writing – review and editing. BI: methodology, validation, resources, writing – review and editing, and funding acquisition. MB: conceptualization, methodology, validation, resources, supervision, investigation, formal analysis (MRI data), writing – review and editing, and funding acquisition. AB: conceptualization, methodology, validation, resources, supervision, investigation, data curation, project administration, formal analysis (neuroimaging data), writing – original draft, writing – review and editing, and funding acquisition. All authors contributed to the article and approved the submitted version.

## Funding

This research was supported by the following grants: National Institutes of Health grant AG058748, AG072328, AG074330, AG034570, AG062542, AG044292, and F31AG063428. MB was supported by the Deutsche Forschungsgemeinschaft (DFG, German Research Foundation; Project ID 425899996 – SFB 1436 Project A08) and by the German Federal Ministry of Education and Research (BMBF, funding code 01ED2102B) under the aegis of JPND. MR data were collected at the Henry H. Wheeler, Jr. Brain Imaging Center, which receives support from the National Science Foundation through their Major Research Instrumentation Program, award number BCS-0821855.

## Conflict of interest

The authors declare that the research was conducted in the absence of any commercial or financial relationships that could be construed as a potential conflict of interest.

## Publisher’s note

All claims expressed in this article are solely those of the authors and do not necessarily represent those of their affiliated organizations, or those of the publisher, the editors and the reviewers. Any product that may be evaluated in this article, or claim that may be made by its manufacturer, is not guaranteed or endorsed by the publisher.
