# Supplementary material for: Interactive effects of locus coeruleus structure and catecholamine synthesis capacity on cognitive function
Source: Front Aging Neurosci. 2023 Sep 6;15:1236335. doi: 10.3389/fnagi.2023.1236335 (PMC10516288; doi:10.3389/fnagi.2023.1236335)
Supplement: Supplementary file 1 [file Data_Sheet_1.docx]

Supplementary Material

**Interactive effects of locus coeruleus structure and catecholamine synthesis capacity on cognitive function**

Hsiang-Yu Chen*, Jourdan H. Parent, Claire J. Ciampa, Martin J. Dahl, Dorothea Hämmerer, Anne Maass, Joseph R. Winer, Renat Yakupov, Ben Inglis, Matthew J. Betts and Anne S. Berry

*Corresponding author: Hsiang-Yu Chen (hsiangyuchen@brandeis.edu)

1. **Analyses and results of the PLSC analysis on the datasets of LC-MRI contrast ratios and [^18^F]FMT PET Ki_vis_ at the voxel-wise whole brain level or within 36 regions of interest.**

An equivalent PLSC analysis was applied to the correlation matrix between LC-MRI contrast ratios and [^18^F]FMT Ki_vis_ measures at the voxel-wise whole brain level or between LC-MRI contrast ratios and [^18^F]FMT Ki_vis_ within 36 ROIs (Desikan et al., 2006). The SVD is formulated as follows.

$SVD[R_{(LC-MRI, FMT)}]=U_{FMT}SV_{LC-MRI}$ (Eq. S1a)

${LV}_{LC-MRI}={X_{LC-MRI}V}_{LC-MRI}$ (Eq. S1b)

${LV}_{FMT}={Y_{FMT}U}_{FMT}$ (Eq. S1c)

where *R* indicates Pearson’s correlation matrix between LC-MRI contrast ratios and [^18^F]FMT Ki_vis_ at the voxel-wise whole brain level or between LC-MRI contrast ratios and [^18^F]FMT Ki_vis_ within Densikan’s 36 ROIs (Eq. S1a) subjected to *SVD*. *U* and *V* are the singular vectors (the saliences) that represent the profiles of LC-MRI contrast and [^18^F]FMT weights at the voxel-wise or ROI level, respectively, to best characterize *R*.

Based on the matrix decomposition, a latent variable (*LV*) was extracted to denote a linear combination of each original dataset (i.e., *X_LC-MRI_* or *Y_FMT_*) and its correspondingly singular vector (i.e., *V_LC-MRI_* or *U_FMT_* in Eq. S1b or S1c). The association between the two latent variables optimally expresses (in the least squares sense) the pattern of interindividual differences in LC-MRI contrast ratios that shares the largest amount of variance with interindividual differences in overall [^18^F]FMT Ki_vis_ at voxel-wise or ROI level. We found no association between the LC-MRI contrast ratios and [^18^F]FMT Ki_vis_ at the voxel-wise or ROI level (*ps* > 0.09)

1. **Analyses and results of the piecewise regression on the latent variables of LC-MRI contrast ratios and [^18^F]FMT PET Ki_vis_ extracted by PLSC.**

A piecewise regression was performed to determine a breakpoint in the association between the LC structural integrity and LC catecholamine synthesis capacity across our 19 participants (9 young adults: 20-29 years [mean ± SD = 24.78 ± 2.21];10 older adults: 65-84 years [mean ± SD = 77.4 ± 5.76]). Although we did find a breakpoint (-1.24 in the LC-MRI contrast ratios; dashed line in Fig. S1), both segments showed positive trend-wise associations (correlation coefficient βs = 0.19 and 2.21) and were not clustered by age groups (Fig. S1).


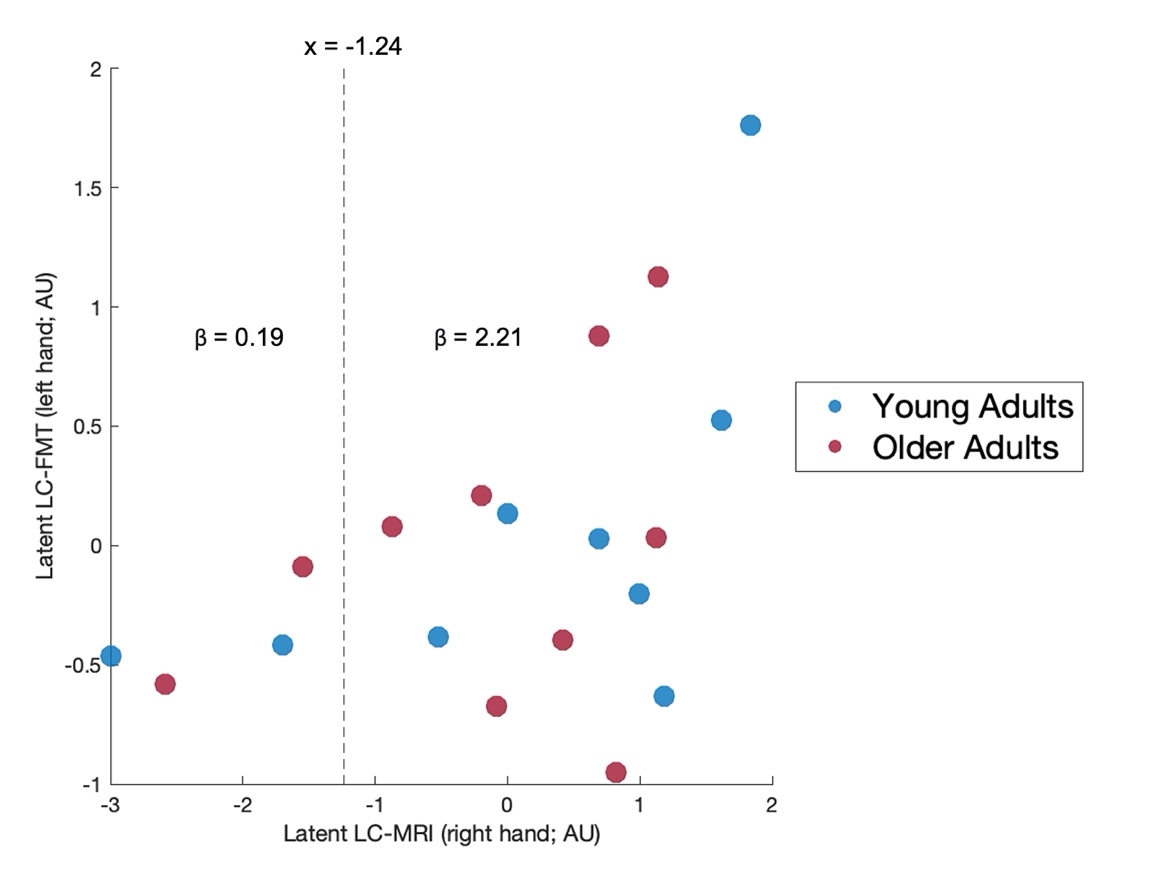


**Fig. S1. Piecewise regression results in 19 participants.** The breakpoint at x = -1.24 on the latent LC-MRI variable was determined, but both segments showed positive trend-wise associations between LC-MRI and LC-FMT (correlation coefficient βs = 0.19 and 2.21) and did not suggest associations between LC-MRI and LC-FMT measures differed based on age groups.
